# Swept-Source OCT Angiography Features in Patients after Macular Hole Surgery

**DOI:** 10.3390/jpm12091493

**Published:** 2022-09-13

**Authors:** Sunjin Hwang, Min-Ho Kang, Mincheol Seong, Heeyoon Cho, Yong-Un Shin

**Affiliations:** 1 Department of Ophthalmology, Hanyang University Guri Hospital, Hanyang University College of Medicine, Guri 11923, Korea; 2 Byers Eye Institute, Stanford Medical School, Stanford, CA 94303, USA

**Keywords:** full-thickness macular hole, optical coherence tomography angiography, choriocapillaris flow void

## Abstract

This study aimed to compare findings of optical coherence tomography (OCT) angiography (OCTA) between eyes with nearly recovered and partially recovered outer retina after full-thickness macular hole (FTMH) surgery and to identify OCTA findings associated with visual acuity. We retrospectively reviewed 30 patients who underwent surgery for idiopathic FTMH. Swept-source OCT (SS-OCT) and OCTA were performed preoperatively and at three and six months postoperatively. Subgroups were divided according to the integrity of the external limiting membranes and ellipsoid zones postoperatively. Correlations of best-corrected visual acuity six months postoperatively with SS-OCT and OCTA measurements were analyzed. There was no difference in preoperative retinal or choroidal vascular index on OCTA between the nearly and partially recovered groups. Six months postoperatively, the choriocapillaris flow-void area was significantly higher in the partially recovered group than in the fellow eye. The nearly recovered group showed better choroidal vascular flow, and vision at six months postoperatively correlated with the ratio of the operated eye’s choriocapillaris flow-void area to the fellow eye. Sufficient choriocapillaris flow on OCTA is associated with better outer retinal recovery and visual outcomes after macular hole surgery.

## 1. Introduction

Full-thickness macular hole (FTMH) is a disease that involves a defect in the fovea from the internal limiting membrane (ILM) to the outer segment of the photoreceptor layer. Vitrectomy is the treatment of choice to induce hole closure by relieving the traction force of the vitreous [1,2,3]. Idiopathic macular hole (MH) surgery has a success rate of approximately 90–100% [4,5,6] Optical coherence tomography (OCT) produces high-resolution retinal images and has become a requisite tool for the diagnosis of FTMH [7,8,9]. OCT-based studies use prognostic factors affecting surgical outcomes, including the minimum linear diameter of MH, basal hole diameter, and properties of the outer retina [10,11,12,13,14]. Some studies have reported that anatomical changes in the choroid may play a role in the development of MH [15,16,17,18].

Optical coherence tomography angiography (OCTA) is a new and noninvasive method for imaging each retinal capillary layer, including the superficial capillary plexus (SCP), deep capillary plexus (DCP), choriocapillaris (CC), and foveal avascular zone (FAZ) [19]. Previous studies have reported on the vascular layer of the retina and choroid in MH patients using OCTA [19,20,21]. However, studies have rarely reported on the relationship between the visual prognosis and the chorioretinal vasculature after MH surgery using OCTA. 

This study aimed to analyze chorioretinal thickness, retinal capillary vessel density, and choriocapillaris flow-void area before and after MH surgery using swept-source OCT (SS-OCT) and OCTA and to investigate factors related to postoperative visual outcomes.

## 2. Materials and Methods

### 2.1. Study Design

This was a retrospective case study of 30 patients who had undergone pars plana vitrectomy (PPV) for an idiopathic MH and follow-up examinations six months postoperatively. All procedures were performed at the Hanyang University Guri Hospital from May 2018 to April 2020. This retrospective study was approved by the Institutional Review Board of Hanyang University Guri Hospital, Gyunggi-do, Korea, which waived the requirement for informed consent. All research adhered to the Declaration of Helsinki (approval number: 2021-12-016), and the data were accessed anonymously.

### 2.2. Ophthalmic Examination

According to the International Vitreomacular Traction Study Group [1], patients with an idiopathic MH with a minimum linear diameter (MLD) of 250–400 μm and no vitreomacular traction were included in this study. The exclusion criteria included MHs with high refractive error (spherical equivalent >6 diopters), an axial length of more than 26.0 mm, trauma, intraocular inflammation, or retinal vasoproliferative disorders; eyes with other retinal diseases such as age-related macular degeneration, diabetic retinopathy, retinal vascular occlusion, or retinal and choroidal inflammatory disease; corneal opacity or glaucoma; history of previous vitrectomies; poor quality SS-OCT and OCTA measurements, including measurement errors such as segmentation errors or image quality scores lower than 45; and patients with a follow-up duration less than six months postoperatively. Best-corrected visual acuity (BCVA), refractive error (spherical equivalent), and axial length of both eyes were measured preoperatively. BCVA was measured with a Snellen chart, and the decimal value was converted to the logarithm of the minimal angle of resolution units (logMAR) for statistical analysis. The spherical equivalent was measured with an autorefractometer, and axial length was determined using an IOL Master (Carl Zeiss Meditec, Dublin, CA, USA). All patients underwent SS-OCT and SS-OCTA (DRI OCT-1 Triton^®^, Topcon Corporation, Tokyo, Japan), and all evaluations were performed before as well as three and six months after the surgery.

### 2.3. Subgroup Analysis

**Figure 1:**
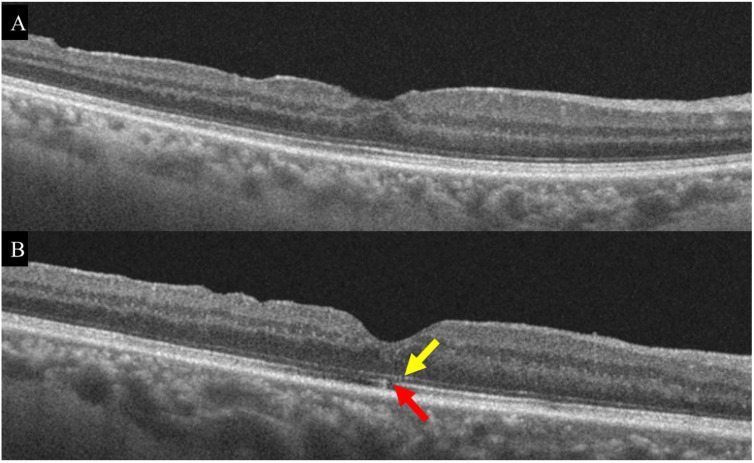
OCT images 6 months after MH surgery. The ELM and EZ lines on OCT maintained continuity without interruption. In this case, the patient was assigned to the nearly recovered group (**A**). When either the ELM (yellow arrow) or EZ (red arrow) line was broken, patients were assigned to the partially recovered group (**B**). ELM, external limiting membrane; and EZ, ellipsoid zone.

We divided patients into two groups—the nearly recovered outer retina or partially recovered outer retina groups—based on the integrity of the external limiting membrane (ELM) and ellipsoid zone (EZ) line continuity [22]. If both lines on OCT were patent without interruptions, they were designated as nearly recovered. If either line was broken, it was designated as partially recovered (Figure 1).

### 2.4. Surgical Procedures

Two experienced surgeons (Y.U.S and H.C) performed standard 23- or 25-gauge PPV with ILM peeling and 20% sulfur hexafluoride gas tamponade in all cases. ILM peeling was performed symmetrically around the fovea after staining with indocyanine green. Patients maintained a facedown position for a week. Patients with a clear lens underwent MH surgery only; otherwise, they underwent MH and cataract surgeries. 

### 2.5. OCT Image Acquisition

**Figure 2:**
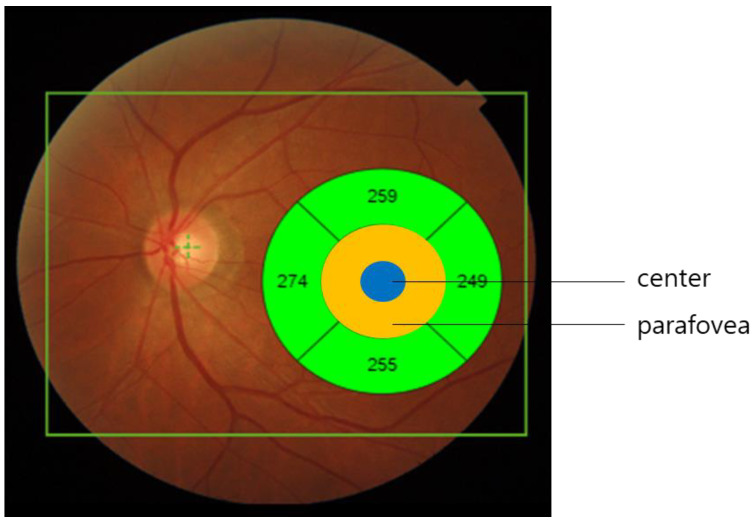
The inner and outer rings with a diameter of 1 and 3 mm around the fovea, respectively, were considered for evaluation. The parafoveal retinal thickness, parafoveal RNFL thickness, and parafoveal GC-IPL thickness were calculated as the average value of outer rings in each layer.

Patients underwent SS-OCT examination before and three and six months after MH surgery. SS-OCT used a 1050-nm wavelength, a speed of 100,000 A-scans per second, an axial resolution of 8 μm, and a transverse resolution of 20 μm. Every patient received a 12 mm × 9 mm three-dimensional wide scan with 256 B-scans, and each scan comprised 512 A-scans (512 A-scans for each of the 256 B-scans). SS-OCT and OCTA images were interpreted using an image viewer (IMAGEnet 6, version 1.28.17646; Topcon Corp., Tokyo, Japan). Central subfield retinal thickness (CSRT), retinal nerve fiber layer (RNFL) thickness, ganglion cell-inner plexiform layer (GC-IPL) thickness, and choroidal thickness were automatically measured in five quadrants (superior, temporal, inferior, nasal, and central) based on a modified Early Treatment Diabetic Retinopathy Study (ETDRS) grid, where the foveal center was automatically placed. The inner (1 mm) and outer rings (3 mm) around the fovea were considered for evaluation (Figure 2). Parafoveal RNFL thickness, GC-IPL, and total thickness were averaged from the values of the outer rings in each layer. In case of segmentation errors, manual modifications were performed by two examiners (S.J.H. and Y.U.S.), and the average values were used. The thickness of the central EZ retinal pigment epithelium (RPE) was also measured by the two examiners and averaged to quantify the status of the outer retina.

### 2.6. OCTA Image Acquisition

SS-OCT applied an eye-tracking system during acquisition and performed proprietary ratio analysis for angiographic processing. The angiography scan covered a 4.5 × 4.5 mm area of the macula, and automated layer segmentation was performed using the instrument’s software IMAGEnet 6 version 1.28.17646 (Topcon Corp., Tokyo, Japan). The SCP layer started at the ILM, and an acceptable thickness was selected that contained the ganglion cell layer between 3 μm below the ILM and 15 μm below the interior boundary of the inner plexiform layer (IPL). DCP contained the capillaries in a layer between 15 μm below the interior boundary of IPL and 70 μm below the interior boundary of IPL. The choriocapillaris comprises capillaries in a layer from the interior boundary of Bruch’s membrane (BM) to 10.4 μm below the BM. 

### 2.7. OCTA Image Analysis

**Figure 3:**
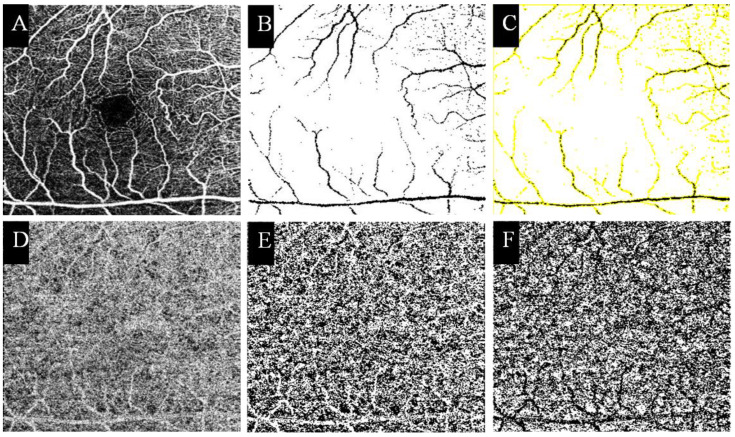
Measurement of the CCP vessel density using the Fiji software. SCP layer images from OCTA (**A**). The image was thresholded, and only large vessels are shown (**B**). We subsequently created (**B**) a masking tool to prevent projection artifacts (**C**). CCP layer from OCTA (**D**). The image was binarized using Otsu’s local-threshold method (**E**). Subsequently, the image (**E**) was masked by another image (**C**) to remove the influence of the large vessel on the choroid (**F**). SCP, superficial capillary plexus; CCP, choriocapillaris.

En face images of the SCP, DCP, and choriocapillaris were exported based on the automated layer segmentation of the OCT software. A quantitative analysis of each OCTA image was performed using the image-processing ImageJ-based software Fiji version 1.51 w (National Institutes of Health, Bethesda, MD, USA). Vessel density (VD) was calculated from the binarized images to quantify the percentage of occupied flow (displayed as white) in the total image area [23]. En face images of SCP and DCP were binarized with Phansalkar local thresholding, and the percentage of VD (displayed as white in the analyzed area) in a 4.5 × 4.5 mm area was calculated using Fiji. The percentage of VD (total vascular area/total measured area × 100) was defined as the fraction of the area filled by vessel lumens in the selected area. Because relatively large retinal vessels would cast shadows on the choriocapillaris due to blood absorption, we masked the large vessels over the choriocapillaris image to remove the influence of the overlying retinal vessels from the subsequent calculation of the flow void [24]. Subsequently, the en face image of the choriocapillaris was binarized with Otsu local thresholding because the Phansalkar local binarization threshold had statistically significant differences compared with various other binarization methods in evaluating the choriocapillaris [23]. The density of the flow voids was measured, excluding the large vessel area. The process was automated using the Fiji software macros (Figure 3). The center was defined as a circle with a radius of 1.0 mm centered at the midpoint of the OCTA scan image, and the total was defined when measuring the entire 4.5 × 4.5 mm area of the OCTA scan. The SCP layer was used to evaluate the FAZ area, which was measured manually using a built-in software tool. Two readers (S.J.H. and Y.U.S.) checked all OCT and OCTA images and modified the segmentation errors before processing the image and excluded images with severe artifacts. The measurement data obtained by the two examiners were averaged and analyzed. We measured intraclass correlation coefficients and assessed the agreement between these measurements.

### 2.8. Statistical Analysis

Statistical analyses were performed using SPSS Statistics software version 22.0 (IBM Corp., Armonk, NY, USA). All data were presented as mean ± standard deviation, and a *p*-value < 0.05 was considered statistically significant. We divided MH patients into two groups based on the status of the EZ-RPE layer and compared the groups with the unaffected eye. The Wilcoxon signed-rank test was used to compare the differences between the MH and unaffected eyes in each group and the differences in the baseline and postoperative parameters. The Mann-Whitney test was used to compare the nearly and partially recovered outer retina groups. 

The ratio of EZ-RPE thickness and choriocapillaris flow void between the MH and unaffected eyes was measured (EZ-RPE thickness_MH/F ratio_ = EZ-RPE thickness of the MH eye/EZ-RPE thickness of the unaffected eye). We used Spearman’s correlation coefficient to measure the strength of the linear relationship between postoperative BCVA and other variables.

## 3. Results

### 3.1. Overall Clinical Characteristics

Thirty eyes of 30 different patients with unilateral idiopathic FTMH were included in this study. Twelve eyes were in the nearly recovered group, and 18 eyes were in the partially recovered group. Table 1 presents the demographic data. The mean age of the enrolled patients was 61.83 ± 10.81 years. Fifty percent of patients were male. The mean baseline logMAR BCVA was 0.61 ± 0.43 in eyes with MH. The nearly recovered and partially recovered groups showed no difference in preoperative BCVA (logMAR BCVA, 0.44 ± 0.22 vs. 0.72 ± 0.50; *p* = 0.16). However, the nearly recovered group had significantly better visual acuity six months postoperatively than that of the partially recovered group (logMAR BCVA, 0.15 ± 0.26 vs. 0.54 ± 0.38; *p* = 0.005). Two investigators reviewed all OCT and OCTA images and manually adjusted segmentation errors. The repeatability of all measurements by each investigator was high (intraclass correlation coefficient [ICC] > 0.9) for each reader. Reproducibility between the two readers was also high (ICC > 0.9). 

### 3.2. Changes in OCT and OCTA Parameters after MH Surgery

When comparing the preoperative and six-month postoperative parameters, CSRT, parafoveal retinal thickness, and central and parafoveal RNFL thickness decreased significantly six months postoperatively (CSRT, *p* = 0.002; parafoveal RT, *p* = 0.001; central RNFL thickness, *p* < 0.001; parafoveal RNFL thickness, *p* < 0.001). Choroidal thickness was significantly thinner at the center (*p* = 0.049) six months postoperatively than preoperatively. The FAZ area significantly decreased from 0.402 ± 0.08 mm^2^ to 0.265 ± 0.090 mm^2^ three months postoperatively (*p* < 0.001) and to 0.251 ± 0.007 mm^2^ six months postoperatively (*p* < 0.001). The SCP VD increased in the center area six months postoperatively (*p* = 0.003), but the DCP VD did not significantly change compared with its preoperative state. The choriocapillaris flow void decreased six months postoperatively in both the center and total area (50.07 ± 3.35% to 48.05 ± 3.88%, *p* = 0.007; and 54.25 ± 1.04% to 52.36 ± 1.05%, *p* = 0.031, respectively) (Table 2). 

There were no significant differences in any of the OCTA parameters between the nearly and partially recovered groups preoperatively (*p* > 0.05 in all OCTA parameters). Six months after MH surgery, in the nearly recovered group, the center and total area of SCP VD (*p* = 0.018, 0.042) and center area of DCP VD (*p* = 0.018) significantly increased. The FAZ area was smaller postoperatively (*p* = 0.015) than was preoperatively. The choriocapillaris flow void in the center area (*p* = 0.018) significantly decreased six months postoperatively compared to its preoperative state. In the partially recovered group, the center area of the SCP VD (*p* = 0.026) and DCP VD (*p* = 0.045) significantly increased six months postoperatively, and the FAZ area size (*p* = 0.009) decreased; however, the choriocapillaris flow void was not significantly different six months postoperatively than preoperatively.

**Figure 4:**
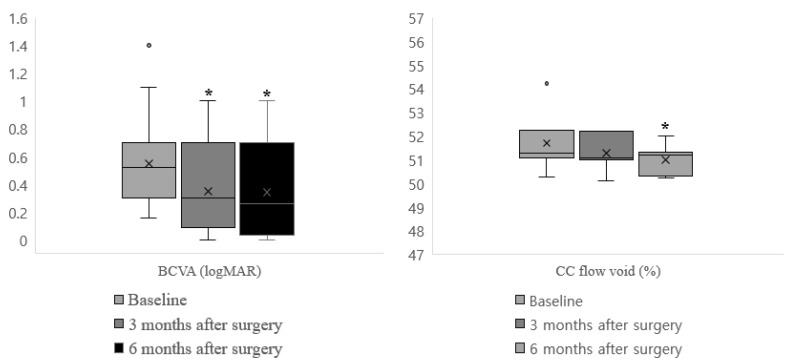
Box-plot graph of BCVA (logMAR) and CC flow void of MH patients. Preoperative data and data at three and six months were compared. BCVA significantly improved three and six months compared to preoperative data (*p* < 0.001). Total area of CC flow void decreased three months postoperatively, yet this difference was not statistically significant (*p* = 0.81). However, choriocapillaris flow restored significantly 6 months postoperatively (*p* = 0.031). * *p*-value < 0.05.

**Figure 5:**
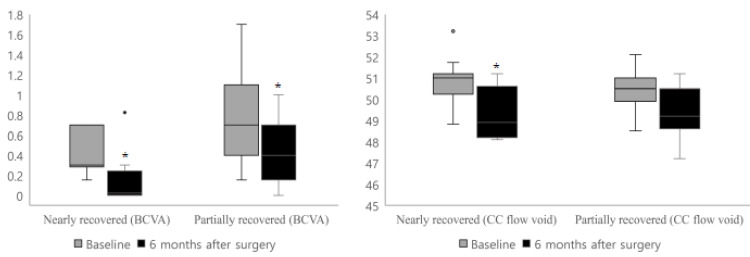
Box-plot graph of BCVA (logMAR) and CC flow void. Preoperative and six months postoperative data of the nearly recovered and partially recovered groups was compared. Compare to preoperative values, BCVA significantly improved six months postoperatively (*p* = 0.005). In the nearly recovered group, the center area of the CC flow void decreased significantly after six months (*p* = 0.018). However, the partially recovered group showed no significant difference in CC flow void six months postoperatively compared to preoperative values (*p* = 0.249). * *p*-value < 0.05.

BCVA improved significantly three and six months postoperatively compared with preoperative values (*p* < 0.001). The total area of the CC flow void decreased three months postoperatively, but the difference was not statistically significant (*p* = 0.81). However, choriocapillaris flow was significantly restored six months postoperatively compared to preoperative values (*p* = 0.031) (Figure 4). The OCT and OCTA parameters, including the retinal and choroidal thickness, VD, choriocapillaris flow void, and FAZ area, did not significantly differ between three and six months postoperatively (Figure 5).

### 3.3. Comparison of OCT and OCTA Parameters between the Unaffected Eye and MH at Six Months Postoperatively

#### 3.3.1. MH Eye in the Nearly Recovered Group versus the Unaffected Eye

The retinal, RNFL, and GC-IPL thicknesses in the central subfield area in the MH eye of the nearly recovered group were higher than those of the unaffected eye (286.63 ± 25.77 µm vs. 224.80 ± 26.57 µm, *p* = 0.005; 19.81 ± 5.56 µm vs. 5.50 ± 2.99 µm, *p* = 0.005; and 72.27 ± 13.83 µm vs. 41.10 ± 10.20 µm, *p* = 0.005, respectively). EZ-RPE thickness was lower in the MH eye in the nearly recovered group than in the unaffected eye (41.54 ± 5.75 µm vs. 49.10 ± 3.24 µm, *p* = 0.008). However, the central and parafoveal choroidal thicknesses were not significantly different between eyes (*p* = 0.333 and *p* = 0.139, respectively) (Table 3). SCP and DCP VD in the central area were significantly larger than those in the unaffected eye (*p* = 0.005, both). The area of the choriocapillaris flow void was not significantly different between eyes. The FAZ area was smaller in the MH eye in the nearly recovered group than in the unaffected eye (*p* = 0.037) (Table 4).

#### 3.3.2. MH Eye in the Partially Recovered Group versus the Unaffected Eye

In the OCT parameter analyses, the partially recovered group showed similar results to the nearly recovered group. The retinal (226.06 ± 64.19 µm vs. 219.43 ± 18.51 µm, *p* < 0.001), RNFL (14.33 ± 6.73 µm vs. 5.18 ± 3.95 µm, *p* = 0.035), and GC-IPL thicknesses in the center (55.06 ± 22.63 µm vs. 37.68 ± 5.91 µm, *p* = 0.011) in the MH eye in the partially recovered group were higher than that in the unaffected eye. EZ-RPE thickness (29.61 ± 15.06 µm vs. 49.00± 2.98 µm, *p* = 0.039) was thinner in the MH eye in the partially recovered than that in the unaffected eye. Moreover, the central and parafoveal choroidal thickness showed no difference between eyes (*p* = 0.22) (Table 3).

**Figure 6:**
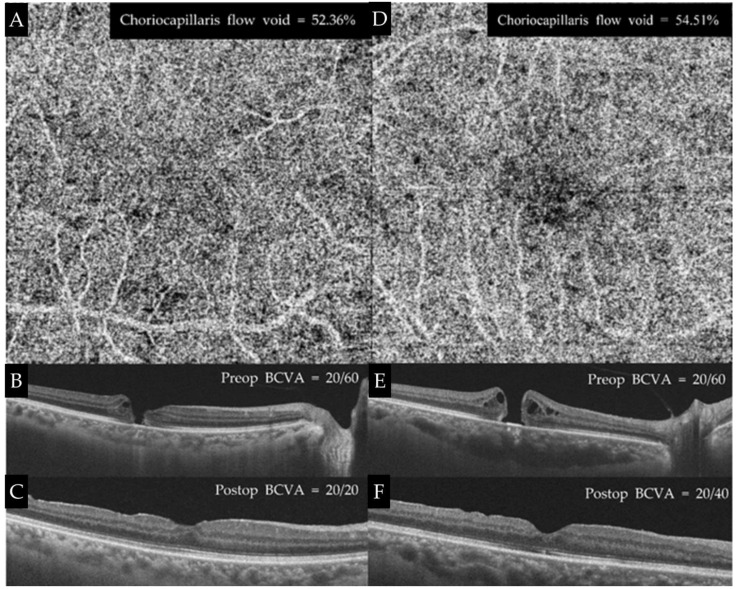
Comparison between the nearly recovered and partially recovered groups. A 63-year-old man showed anatomic and functional restoration after macular hole surgery. Choriocapillaris flow void was 52.36% postoperatively (**A**). His vision was 20/60, and OCT revealed FTMH (**B**). Six months postoperatively, his vision was restored to 20/20, and there was no discontinuity in both the EZ and ELM line (**C**). A 73-year-old woman underwent macular hole surgery due to FTSSMH (**D**), and her preoperative BCVA was 20/60. Some discontinuity in the EZ line was left (**E**). Postoperatively, her vision was 20/40, and choriocapillaris flow void was 54.51% (**F**).

SCP, VD, and DCP VD in the center were significantly larger in the MH eye in the partially recovered group than in the unaffected eye (*p* = 0.015 and *p* = 0.026, respectively). The total area of the choriocapillaris flow void was significantly larger in the MH eye in the partially recovered group than in the unaffected eye (54.11 ± 1.01% and 51.73 ± 1.15%, respectively, *p* = 0.022), but there was no significant difference in the central area choriocapillaris flow void between eyes (*p* = 0.374). The FAZ area was smaller in the MH eye of the partially recovered group than in the unaffected eye (*p* = 0.015) (Table 4 and Table 5, Figure 6).

### 3.4. Correlation Analyses of the OCT and OCTA Parameters and BCVA

**Figure 7:**
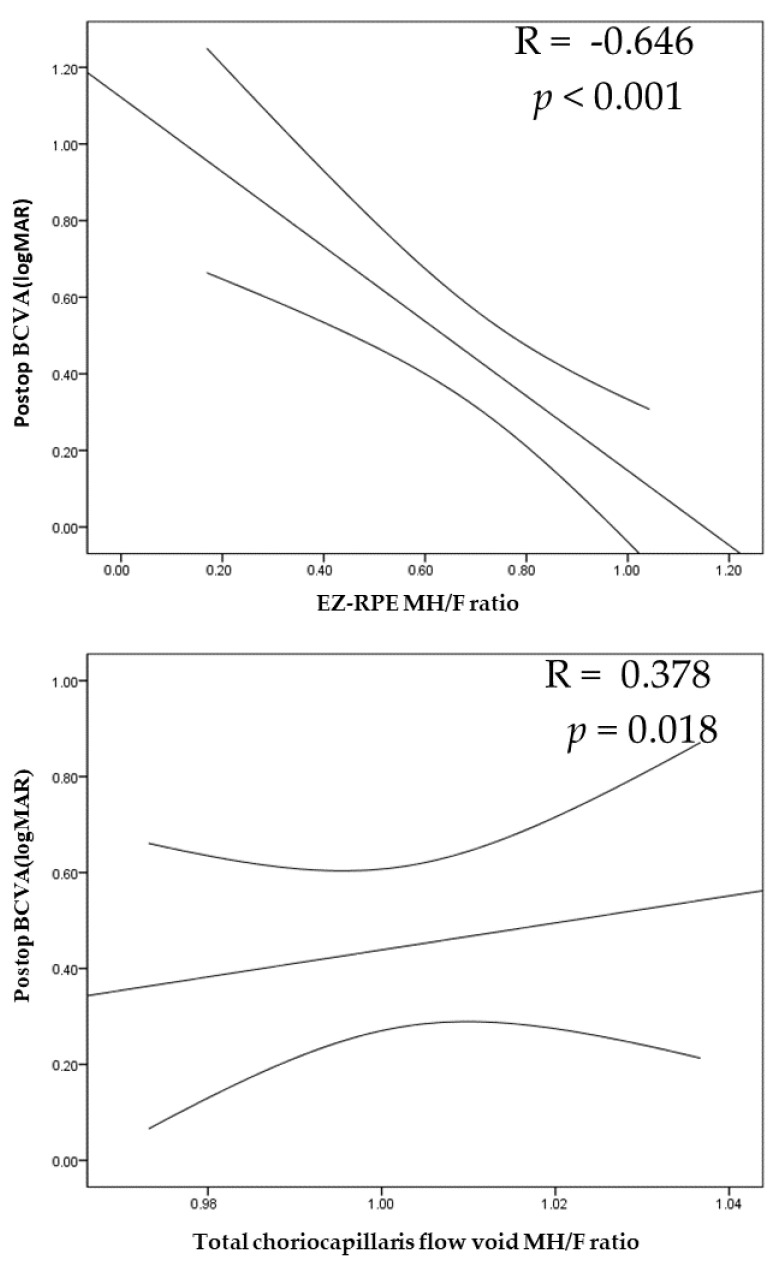
Correlation of postoperative BCVA with the EZ-RPE MH/F and total CC flow void MH/F ratios. The curved lines indicate a 95% confidence interval of the linear regression line. EZ-RPE MH/F ratio, ellipsoid zone to retinal pigment epithelium distance in the MH/unaffected eye; total CC flow void MH/F ratio, total choriocapillaris flow void of the MH/unaffected eye.

Correlation analyses were performed to further evaluate which OCT and OCTA parameters correlated with postoperative BCVA (Table 6). Postoperative BCVA significantly correlated with postoperative EZ-RPE thickness_MH/F ratio_ (R = −0.646, *p* < 0.001) and the postoperative choriocapillaris flow void_MH/F ratio_ (R = 0.378, *p* = 0.018). However, postoperative BCVA showed no correlation with retinal and choroidal thickness at the last follow-up (*p* = 0.086 and *p* = 0.574, respectively) (Figure 7).

## 4. Discussion

This study investigated structural and vascular changes in idiopathic FTMH pre- and postoperatively using SS-OCT and SS-OCTA. We compared chorioretinal structure and microvasculature by dividing the eyes into two groups based on the degree of outer retinal recovery, which is an important prognostic factor for visual recovery after MH surgery. We found that the choriocapillaris flow void appeared to be related to the degree of recovery of the outer retinal layer. Furthermore, our study indicated that EZ-RPE thickness and small choriocapillaris flow voids were associated with better visual acuity after MH surgery.

Many studies have reported inner retinal thinning on OCT after MH surgery, in which the ILM peeling technique leads to higher anatomical closure and lower recurrence rates [3]. However, the thickness of the inner retinal layer adjacent to the ILM could be altered by surgery [25]. Baba et al. [26] and Ohta et al. [27] reported a decrease in the ganglion cell complex thickness after MH surgery. RNFL and ganglion cell layer (GCL) thinning after MH surgery, possibly due to mechanical damage to the GCL or Muller cells in the GCL induced by ILM peeling, have been reported in another study [28]. In our study, the RNFL and GC-IPL thicknesses significantly decreased at three and six months postoperatively compared with baseline values, which is consistent with the findings of previous studies.

Takamura et al. [29] reported a significant positive correlation between central retinal thickness one month after MH surgery and visual acuity one year after MH surgery. Central retinal thickness at 3, 6, and 12 months was not significantly correlated with visual acuity, suggesting that increased central retinal thickness in the early postoperative period may result in a better visual prognosis because of the higher degree of filling with neuronal tissue. However, Wilczynski et al. showed a strong correlation between postoperative BCVA and CRT three months postoperatively. In our study, CSRT at three and six months postoperatively was unrelated to visual outcomes. However, EZ-RPE thickness, which is highly relevant to outer retinal recovery, strongly correlated with visual acuity prognosis, confirming that outer retinal recovery is important for visual acuity recovery [11,14,18].

In the present study, when analyzing choroidal thickness preoperatively, the OCT parameter and choroidal thickness of the MH eye was thinner than that of the unaffected eye. Zhang et al. [16] reported that the subfoveal choroidal thickness of patients with unilateral MH (202.78 ± 58.90 μm) was thinner than that of normal individuals (272.38 ± 57.27 μm). In another study, when comparing the choroidal thicknesses between eyes in patients with MH, that of the unaffected eye was 206.82 ± 67.09 μm, whereas that of the normal eye was 248.88 ± 63.10 μm [15]. A previous study reported that unaffected eyes that had a thinner choroid than normal eyes and had a six-fold higher risk of MH formation [30], Indicating that patients with thinner choroids may be prone to idiopathic MH. Karkhaneh et al. [31] reported a relationship between choroidal thickness and basal MH diameter but did not reveal an association between choroidal thickness and visual outcomes. In our study, there was no association between choroidal thickness and visual prognosis. Since the mechanism of MH occurrence and surgical site include the vitreoretinal face and ILM, choroidal thickness is thought to have little effect on MH recovery. 

In this study, MH patients showed structural and microvascular changes on OCTA postoperatively. When analyzing the FAZ area of MH patients using OCTA, there was a measured enlargement due to centrifugal tractional force on MH preoperatively, which can be interpreted as a loss of the retinal capillary plexus. Because the tractional force is released after ILM peeling, a reduction in the FAZ area was found due to the centripetal dragging of retinal tissues [32]. In previous studies, the FAZ area reportedly changed preoperatively to postoperatively from 0.45 ± 0.14 mm^2^ to 0.25 ± 0.08 mm^2^ [33], 0.39 ± 0.07 mm^2^ to 0.24 ± 0.07 mm^2^ [34] and 0.425 ± 0.120 mm^2^ to 0.232 ± 0.120 mm^2^, respectively [35], demonstrating results similar to those in our study (0.402 ± 0.08 mm^2^ to 0.251 ± 0.01 mm^2^), with the FAZ area getting smaller and the SCP and DCP increasing postoperatively. In normal patients, the FAZ area and central retinal thickness are inversely correlated [36,37]. The same relationship exists after MH surgery [33,35]. In our study, not only the CSRT but also the RNFL and GC-IPL thicknesses negatively correlated with the FAZ area.

Sufficient choroidal blood flow is thought to be related to outer retinal recovery and improved vision. Studies have been conducted on the relationship between MH and choriocapillaris flow using OCTA. Wilczyński et al. [34] found that the parafoveal choriocapillaris VD increased after MH surgery using SD-OCTA. Teng et al. [20] reported that choriocapillaris circulation in the parafoveal area increased postoperatively. Other studies have investigated the relationship between other retinal diseases and choriocapillaris flow using OCTA. Hong et al. [38] found that choriocapillaris flow could be related to recovery of the outer retinal layer, which might predict visual outcomes after retinal detachment surgery. Nesper et al. [39] also found that choriocapillaris nonperfusion was associated with visual compromise in reticular pseudodrusen. Our study found a statistical decrease in choriocapillaris flow void in patients with MH postoperatively (50.07 ± 3.35 to 48.05 ± 3.88, *p* = 0.007) using SS-OCTA, indicating an increase in choriocapillaris flow.

Furthermore, we compared the choriocapillaris flow in nearly and partially recovered patients pre- and postoperatively. Although there were no significant differences in choriocapillaris flow between both groups at baseline, the nearly recovered group showed significant improvements in the choriocapillaris flow void six months after MH surgery. However, the partially recovered group showed no recovery. It seems that some mechanisms, such as light toxicity intraoperatively, ICG toxicity, extent of ILM peeling, or surgery time, might affect the recovery of MH, which would lead to differences between the nearly recovered group and partially recovered group. However, the underlying mechanism remains unclear, and further research is needed.

The postoperative recovery of MH begins with the connection of the inner retina and bridge-like glial proliferation, followed by a gradual restoration of the intact photoreceptor body [7]. Retinal blood vessels are important components of the retinal structure and are involved in the development and healing of retinal diseases [40]. Choriocapillaris flow is mandatory to supply oxygen and nutrients to the outer retina [41]. Our results indicate that the recovery state of the outer retina after MH surgery is associated with postoperative choriocapillaris flow void and correlates well with visual outcomes. In the nearly recovered group, choriocapillaris flow void did not differ from that in the unaffected eye. However, the choriocapillaris flow void in the partially recovered group was notably larger than that in the unaffected eye. The blood supply to the retina differs between the outer and inner retinas. RPE, photoreceptor layer, ELM, and outer nuclear layer are supplied by the choriocapillaris, whereas the inner nuclear layer and outer plexiform layer are supplied by the central retinal artery and in part by the choriocapillaris layer to form the DCP. The central retinal artery supplies the nerve fiber and ganglion cell layers to form the SCP [42]. Thus, a well-maintained choriocapillaris can lead to the recovery of the outer retina. There was a difference in the choriocapillaris flow void in the total area (*p* = 0.02) between the partially recovered group and the unaffected eye; however, there was no difference in the central area of the choriocapillaris flow void (*p* = 0.378). It seems that the choriocapillaris of the parafoveal area plays a larger role in restoring the outer retina than the central area, but further research is necessary to confirm these findings. In summary, the nearly recovered group, which revealed better visual acuity postoperatively than the partially recovered group, showed improvements in choriocapillaris flow in the MH eye postoperatively compared with preoperative values, as well as the unaffected eye. This was not accomplished in the partially recovered group. Hence, a better restoration of choriocapillaris flow might lead to sufficient outer retinal recovery and better vision. 

This study had several limitations. First, it was retrospective and had a relatively small sample size, which could have resulted in an insufficient number of subjects for adequate analysis. Second, as the OCT images of the MH rendered some segmentation errors, we manually measured the retinal thickness of the MH. Third, our study only covered medium-sized MH (MLD between 250 and 400 μm), and further investigations of large MH (MLD larger than 400 μm) are needed.

Despite these limitations, our study had several strengths. First, by including patients with a follow-up appointment of six months, we were able to identify changes that occurred over a long period compared with other studies [10,34]. Second, we used SS-OCT, which has a superior resolution than that of SD-OCT because of the longer wavelengths. The 1050-nm wavelength enabled the better visualization of deep layers, including the choriocapillaris, as well as a higher number of more precise scans. However, SS-OCT has a faster scanning speed than SD-OCT, and we were able to easily acquire OCT data from unamenable subjects. In addition, we divided patients into two subgroups according to the extent of their outer retinal recovery, which provided some clues as to which mechanisms could make MH surgery more successful.

## 5. Conclusions

The recovery of the outer retinal layer, which is closely related to visual prognosis after MH surgery, seems to be related to the flow deficit area of the choriocapillaris. The prognosis of MH is affected by several factors; however, evaluating chorioretinal vascular changes using SS-OCTA can help predict visual outcomes after MH recovery.

## Figures and Tables

**Table 1:** Demographics and clinical characteristics of patients.

Characteristics	Total	Outer Retina Status Six Months after Surgery		
		Nearly Recovered	Partially Recovered	*p*-Value
Numbers of patients	30	12	18	
Age (years)	61.83 ± 10.81	62.90 ± 4.70	61.17 ± 13.36	0.808
Gender (male)	15	6	9	0.092
Laterality (right)	15	6	9	0.162
Axial length (mm)	23.48 ± 1.26	23.39 ± 0.79	23.53 ± 1.48	0.555
Baseline BCVA (logMAR)	0.61 ± 0.43	0.44 ± 0.22	0.72 ± 0.50	0.16
Postoperative BCVA (logMAR) (6 months)	0.39 ± 0.39	0.15 ± 0.26	0.54 ± 0.38	0.005
Minimum linear diameter (MLD) of the macular hole (μm)	305.40 ± 118.30	308.25 ± 101.98	301.54 ± 91.58	0.653

Data are presented as *n* (%) or mean ± standard deviation as appropriate. Comparisons between the nearly and partially recovered outer retina groups were performed using the Mann-Whitney test. BCVA, best corrected visual acuity.

**Table 2:** Comparisons between the baseline and postoperative BCVA, OCT and OCT-A parameters.

Variables		Baseline	Three Months after Surgery	Six Months after Surgery	*p*-Value		
					Base vs. 3m	Base vs. 6m	3m vs. 6m
BCVA (logMAR)		0.61 ± 0.43	0.4 ± 0.37	0.39 ± 0.39	0.001	0.001	0.453
Retina thickness (μm)	Center	331.88 ± 92.69	253.91 ± 49.40	249.03 ± 60.28	0.003	0.002	0.624
	Parafovea	335.78 ± 38.08	306.38 ± 26.23	306.31 ± 26.87	0.001	0.001	0.508
RNFL thickness (μm)	Center	50.80 ± 52.57	17.86 ± 9.62	16.41 ± 6.77	<0.001	<0.001	0.838
	Parafovea	45.74 ± 18.54	29.72 ± 3.66	28.53 ± 3.37	<0.001	<0.001	0.476
GC-IPL thickness (μm)	Center	67.16 ± 25.57	55.47 ± 12.48	56.58 ± 11.25	0.012	0.011	0.143
	Parafovea	84.83 ± 11.33	70.22 ± 10.25	69.32 ± 12.79	0.015	0.018	0.215
Choroid thickness (μm)	Center	211.23 ± 67.75	206.39 ± 85.18	192.96 ± 73.17	0.765	0.049	0.289
	Parafovea	189.90 ± 68.58	198.53 ± 78.88	182.22 ± 59.54	0.477	0.085	0.136
EZ-RPE thickness (μm)	Center	n/a	32.08 ± 12.16	34.13 ± 13.57	n/a	n/a	0.346
FAZ area (mm^2^)		0.402 ± 0.08	0.265 ± 0.09	0.251 ± 0.007	<0.001	<0.001	0.612
SCP VD (%)	Center	22.77 ± 3.47	29.43 ± 5.18	30.12 ± 5.60	0.004	0.003	0.173
	Total	47.63 ± 2.27	47.22 ± 1.78	47.06 ± 1.82	0.232	0.943	0.463
DCP VD (%)	Center	24.17 ± 3.22	33.50 ± 6.95	32.07 ± 8.57	0.011	0.227	0.6
	Total	52.64 ± 4.30	52.96 ± 2.68	51.66 ± 1.95	0.957	0.943	0.28
Choriocapillaris flow void (%)	Center	50.07 ± 3.35	48.18 ± 3.44	48.05 ± 3.88	0.027	0.007	0.146
	Total	54.25 ± 1.04	53.18 ± 1.09	52.36 ± 1.05	0.81	0.031	0.117

Data are presented as *n* (%) or mean ± standard deviation as appropriate. *p*-values were calculated using a three-way ANOVA test. The central area was a circle of 1.0 mm radius centered in the middle of the image. The parafovea was the average of the superior, temporal, inferior, and nasal sectors of the outer ring of the ETDRS grid. The total area is × 4.5 × 4.5 mm area of the macula. BCVA, best-corrected visual acuity; RNFL, retinal nerve fiber layer; GC-IPL, ganglion cell-inner plexiform layer; VD, vessel density; SCP, superficial capillary plexus; DCP, deep capillary plexus; CC, choriocapillaris.

**Table 3:** Comparison between the retinal thickness of eyes with full thickness macular holes six months postoperatively and unaffected eyes.

OCT Parameters (μm)	Area	Nearly Recovered Group				Partially Recovered Group		
		Operated Eye	Unaffected Eye	*p*-Value *	Operated Eye	Unaffected Eye	*p*-Value **	*p*-Value ***
Retinal thickness	Center	286.63 ± 25.77	224.80 ± 26.57	0.005	226.056 ± 64.19	219.43 ± 18.51	0.307	0.001
	Parafovea	307.06 ± 15.18	299.57 ± 8.38	0.047	305.86 ± 32.45	305.18 ± 14.90	0.061	0.642
RNFL thickness	Center	19.81 ± 5.56	5.30 ± 3.16	0.005	14.33 ± 6.73	5.18 ± 3.95	0.001	0.035
	Parafovea	28.02 ± 3.57	26.45 ± 4.34	0.074	28.85 ± 3.31	27.20 ± 3.94	0.01	0.34
GCIPL thickness	Center	72.27 ± 13.83	40.70 ± 10.41	0.005	55.06 ± 22.63	37.68 ± 5.91	0.001	0.011
	Parafovea	82.20 ± 10.81	86.32 ± 12.65	0.683	80.79 ± 14.14	87.35 ± 7.21	0.222	0.982
EZ-RPE thickness	Center	41.54 ± 5.75	49.10 ± 3.24	0.008	29.61 ± 15.06	49.00 ± 2.98	0.001	0.039
Choroidal thickness	Center	215.45 ± 60.17	223.90± 71.07	0.333	179.22 ± 78.49	194.31 ± 43.67	0.776	0.22
	Parafovea	205.11 ± 61.77	216.50 ± 59.40	0.139	168.24 ± 55.21	190.93 ± 51.10	0.379	0.22

* Comparisons between the nearly recovered outer retinal group’s MH eyes and unaffected eyes six months after surgery were performed using a Wilcoxon signed-rank test. ** Comparisons between the partially recovered outer retinal group’s MH eyes and unaffected eyes six months after surgery performed using a Wilcoxon signed-rank test. *** Comparisons between the nearly recovered and partially recovered outer retinal groups’ MH eyes six months after surgery performed using a Mann-Whitney test. The central area is a circle of a 1.0 mm radius centered in the middle of the image. The parafovea is the average of the superior, temporal, inferior, and nasal sectors of the outer ring of the ETDRS grid. RNFL, retinal nerve fiber layer; GC-IPL, ganglion cell-inner plexiform layer.

**Table 4:** Comparison between the vessel density of eyes with full thickness macular hole six-months postoperatively and unaffected eyes.

OCT Parameters	Area	Nearly Recovered Group				Partially Recovered Group		
		Operated Eye	Unaffected Eye	*p*-Value *	Operated Eye	Unaffected Eye	*p*-Value **	*p*-Value ***
SCP VD (%)	Center	35.27 ± 2.32	25.17 ± 4.47	0.005	27.04 ± 5.21	23.21 ± 3.86	0.015	0.122
	Total	47.43 ± 1.40	47.07 ± 1.57	0.386	46.82 ± 2.05	47.02 ± 2.07	0.424	0.611
DCP VD (%)	Center	34.20 ± 6.75	22.29 ± 6.40	0.005	31.88 ± 6.20	24.77 ± 6.14	0.026	0.164
	Total	51.14 ± 1.87	50.57 ± 2.04	0.444	51.99 ± 1.99	52.07 ± 2.81	0.374	0.43
Choriocapillaris flow void (%)	Center	48.81 ± 2.90	49.43 ± 2.62	0.386	47.78 ± 3.26	49.13 ± 5.76	0.374	0.311
	total	52.27 ± 0.92	51.94 ± 0.44	0.169	54.11 ± 1.01	51.73 ± 1.15	0.022	0.638
FAZ area size (mm^2^)		0.301 ± 0.964	0.47 2± 0.140	0.037	0.382 ± 0.132	0.496 ± 0.876	0.015	0.109

* Comparisons between the nearly recovered outer retinal group’s MH eyes and unaffected eyes six months after surgery were performed using a Wilcoxon signed-rank test. ** Comparisons between the partially recovered outer retinal group’s MH eyes and unaffected eyes six months after surgery performed using a Wilcoxon signed-rank test. *** Comparisons between the nearly recovered and partially recovered outer retinal groups’ MH eyes six months after surgery performed using a Mann-Whitney test. The central area is a circle of a 1.0 mm radius centered in the middle of the image. The total area is × 4.5 × 4.5 mm area of the macula. VD, vessel density; SCP, superficial capillary plexus; DCP, deep capillary plexus; CC, choriocapillaris.

**Table 5:** Comparison between the vessel density of eyes with the nearly recovered group and the partially recovered group preoperatively and six-months postoperatively.

OCT Parameters	Area	Nearly Recovered Group				Partially Recovered Group		
		Preop.	Postop 6mo.	*p*-Value *	Preop.	Postop 6mo.	*p*-Value **	*p*-Value ***
SCP VD (%)	Center	21.76 ± 2.79	35.27 ± 2.32	0.018	23.55 ± 4.38	27.04 ± 5.21	0.026	0.252
	Total	46.23 ± 2.01	47.43 ± 1.40	0.042	45.72 ± 1.88	46.82 ± 2.05	0.345	0.131
DCP VD (%)	Center	21.43 ± 7.11	34.20 ± 6.75	0.018	26.29 ± 13.47	31.88 ± 6.20	0.045	0.47
	Total	51.16 ± 2.71	51.14 ± 1.87	0.999	50.03 ± 5.19	51.99 ± 1.99	0.463	0.408
Choriocapillaris flow void (%)	Center	50.25 ± 5.95	48.81 ± 2.90	0.018	49.92 ± 9.94	47.78 ± 3.26	0.249	0.681
	total	51.67 ± 0.92	52.27 ± 0.92	0.236	51.00 ± 1.12	54.11 ± 1.01	0.139	0.299
FAZ area size (mm^2^)		0.542 ± 0.0155	0.301 ± 0.964	0.015	0.826 ± 0.130	0.382 ± 0.132	0.009	0.702

* Comparisons between the preoperative and six-month postoperative data of the nearly recovered group using the Wilcoxon signed-rank test. ** Comparisons between the preoperative and six-month postoperative data of the partially recovered group using a Wilcoxon signed-rank test. *** Comparisons between the preoperative data of the nearly recovered group and partially recovered group using a Mann-Whitney test. The central area is a circle of a 1.0 mm radius centered in the middle of the image. The total area is × 4.5 × 4.5 mm area of the macula. VD, vessel density; SCP, superficial capillary plexus; DCP, deep capillary plexus; CC, choriocapillaris.

**Table 6:** Spearman’s rank correlation of optical coherence tomography (OCT) and OCT angiography parameters (MH/F ratio) and postoperative best-corrected visual acuity (logMAR) in the MH group.

Thickness	Center	*p*-Value	Parafovea	*p*-Value
Retina	−0.413	0.086	−0.405	0.064
RNFL	−0.093	0.65	0.386	0.104
GC-IPL	−0.315	0.117	−0.233	0.252
Choroid	−0.116	0.574	−0.144	0.481
EZ-RPE	−0.646	<0.001		
	Center	*p*-value	Total	*p*-value
SCP VD	−0.289	0.191	0.032	0.889
DCP VD	−0.002	0.94	−0.111	0.622
Choriocapillaris flow void	−0.029	0.974	0.378	0.018
FAZ area	0.143	0.536		

## Data Availability

The data supporting the findings of this study are available from the corresponding author upon reasonable request.
